# Comparison of Arterial Stiffness and Strain Measured with Speckle Tracking Carotid Strain Ultrasonography after Radiation and Surgical Treatment for Head and Neck Cancer—A Clinical Trial

**DOI:** 10.3390/diagnostics13193090

**Published:** 2023-09-29

**Authors:** Bengu Depboylu, Aylin Eryilmaz, Hatice Sema Basak, Veli Kirbac, Yesim Basal, Imran Kurt Omurlu, Mustafa Gok

**Affiliations:** 1 Department of Radiation Oncology, Faculty of Medicine, Aydın Adnan Menderes University, Aydın 09010, Türkiye; drbdenizli@gmail.com; 2 Department of Otolaryngology, Faculty of Medicine, Aydın Adnan Menderes University, Aydın 09010, Türkiye; draylineryilmaz@gmail.com (A.E.); haticesemabasak@gmail.com (H.S.B.); kirbacveli@gmail.com (V.K.); yesimdurgun@gmail.com (Y.B.); 3 Department of Biostatistics, Faculty of Medicine, Aydın Adnan Menderes University, Aydın 09010, Türkiye; imran.omurlu@adu.edu.tr; 4 Department of Radiology, Faculty of Medicine, Aydın Adnan Menderes University, Aydın 09010, Türkiye; 5 Department of Health Sciences, Faculty of Medicine and Health, University of Sydney, Sydney, NSW 2050, Australia

**Keywords:** cardiovascular disease, carotid artery stiffness, head and neck cancer, neck dissection, neck irradiation, speckle tracking carotid strain ultrasonography

## Abstract

This study assessed arterial stiffness in head and neck cancer patients using speckle tracking carotid strain ultrasonography (STCS-US). It investigated the impacts of neck irradiation and neck dissection on the arterial stiffness of these patients by comparing their stiffness parameters with those of healthy controls. A total of 101 participants (67 patients and 34 healthy controls) were enrolled in this study. Fifty-two patients received definitive radiation therapy (TD: 60–72 Gy in 30 days) at least two years ago. Participants were grouped into four according to their states of neck irradiation (IR) and neck dissection (ND): Group (IR+/ND−) had 28 patients, Group (IR+/ND+) had 24 patients, Group (IR−/ND+) had 15 patients, and Group (IR−/ND−) had 34 healthy controls. All the participants underwent STCS-US. Arterial stiffness parameters relating to arterial compliance (AC) and elastic modulus (EM) were significantly changed in Group (IR+/ND−) and Group (IR+/ND+) in the transverse plane (*p* < 0.001, *p* < 0.001) and in the longitudinal plane (*p* < 0.001, *p* < 0.001); the change in β-stiffness index (β-SI) was more significant in the transverse plane (*p* = 0.002). Group (IR+/ND+) had significant transverse circumferential (*p* = 0.001) and radial strain parameters (*p* = 0.001). The carotid intimal medial thickness (CIMT) significantly changed in Group (IR+/ND+) compared to controls (*p* = 0.001). Our findings indicate that neck irradiation and neck dissection increase arterial stiffness as single treatments; however, double treatment is associated with a higher increase. Neck irradiation affects strain parameters more than neck dissection alone. The study demonstrated the feasibility and clinical value of the STCS method in assessing arterial stiffness and its potential use in cardiovascular risk assessment for patients with head and neck cancer.

## 1. Introduction

Radiation therapy (RT) is one of the integral treatment modalities for head and neck cancers. A significantly increased risk for atherosclerotic changes in the head and neck area has been confirmed in both animal models and human studies within the radiation field [1,2]. Early studies in which older ionizing irradiation techniques are used report that up to one in four irradiated patients exhibit carotid artery damage leading to stenosis [3]. Pathologically, radiation-therapy-induced atherosclerosis is described as more fibrotic than its inflammatory counterparts in radiation-naive carotid artery samples [4]. Moreover, it differs from other atherogenic risk factors with its limited-to-the-irradiated-area nature from other atherogenic risk factors [5].

Even though many studies have investigated the effect of head and neck radiotherapy on arterial stiffness, few studies have focused on the effect of neck dissection [6,7,8,9,10,11]. Head and neck cancer patients have a high risk for neck metastasis because the neck region is rich in lymphatic drainage. The most critical factor for prognosis in the absence of distant metastasis for these patients is the extension of the nodal involvement in the neck area [12]. In this case, neck dissection is a diagnostic and therapeutic procedure that defines the disease’s extent, further establishing the type of adjuvant oncologic treatment [13].

Various types of neck dissections are performed depending on the patterns of nodal metastases regarding the primary location of the malignancy [14]. Lymphoid tissues between the fasciae of the internal jugular vein and the carotid artery are removed during neck dissection, changing the relationship between the carotid artery and the internal jugular vein. In some neck dissection types, excision of the sternocleidomastoid muscle or ligation and removal of the internal jugular vein might be indicated. In such cases, no barrier other than the skin remains on the carotid artery. These traumatic procedures have been considered to contribute to arterial stiffness to a great extent [15,16].

Definitive RT for locally advanced head and neck cancer involves irradiation of the primary tumor and involved cervical lymph node chain in the vicinity of carotid arteries along their entire trace, exposing them to moderate to high doses of radiation [3,6]. Postoperative radiotherapy or chemoradiotherapy is routinely administered in cases with neck dissection, including entire neck levels in the radiation fields. This approach is sufficient to achieve excellent control rates in undissected levels. Furthermore, modern irradiation techniques and current treatment planning systems enable the delivery of highly selective and accurate doses to different neck regions. Meanwhile, the patient experiences the advantage of reduced morbidity and personalized nodal irradiation. Retrospective studies document a higher prevalence of radiation-induced atherosclerosis in the distal parts of the irradiated internal or external carotid artery [5]. The carotid artery’s stiffness level was higher on the irradiated side of the neck than on the non-irradiated side [7].

Recently, arterial stiffness has been considered an early marker of subclinical atherosclerosis [17]. The factors determining arterial stiffness are structural components of the arterial wall, especially elastin and collagen, vascular smooth muscle tone, and transmural distension pressure [12]. Studies have shown the utility and sensitivity of the speckle tracking carotid strain ultrasound (STCS-US), which has been introduced to clinical practice to evaluate the stiffness parameters multidimensionally by assessing the functional and morphologic changes in the arterial wall via B-mode ultrasound [13,14,16]. Functional changes occur before structural changes, so the STCS-US technique indicates the functional changes caused by atherosclerosis in the arterial wall [18]. This method can measure arterial compliance (AC), arterial distensibility (AD), elastic mode index (EMI), arterial strain, the β-stiffness index (β-SI), and pulse wave velocity (PWV) regarding arterial wall stiffness. In contrast, displacement, strain, and strain rate can be calculated for arterial strain characteristics [13,18].

The definition of AC indicates the absolute change in vessel diameter according to changes in applied pressure. AD represents the relative change in vessel diameter according to changes in applied pressure. The EMI is the alteration in pressure necessary for a theoretical stretch from the resting vessel diameter. β-SI is the ratio of the natural logarithm of systolic/diastolic pressure to the relative change in vessel diameter. The definition of PWV is the pulse wave speed that extends throughout the length of the vessel [13]. Increases in elastic modulus, β-stiffness index, and pulse wave velocity values indicate increased arterial stiffness. As arterial stiffness increases, the capacity of the artery to transform pulsatile stress into continuous stress in the brain increases. It is also a risk factor for cardiovascular events like stroke [6].

Our goal in this study is to assess arterial stiffness in head and neck cancer patients using STCS-US. We investigated the impacts of neck irradiation and neck dissection on the arterial stiffness of these patients by comparing their stiffness parameters with healthy controls.

## 2. Materials and Methods

### 2.1. Study Population

Head and neck cancer patients previously diagnosed and evaluated at the Otorhinolaryngology-Oncology Tumor Board of Aydın Adnan Menderes University Hospital were assessed retrospectively between January 2015 and December 2020. Eligible patients who were older than 18 years, underwent neck dissection, received neck radiotherapy, or both, were invited via telephone call to participate in this study. The patients with nasopharyngeal carcinoma were not invited, as neck dissection was not primarily indicated in their management. Of the 180 eligible patients who were invited, the following were excluded from the study: 40 were dead, 15 declared they had COVID-19 infection, 18 refused to participate in it, 6 moved to another city, and 31 did not respond to the invitation. A further 3 patients were excluded from the study because they could not tolerate the ultrasound procedure. The remaining 67 patients were included in the study, 52 of whom had at least a two-year history of definitive head and neck radiotherapy and 39 of whom had neck dissection.

**Figure 1:**
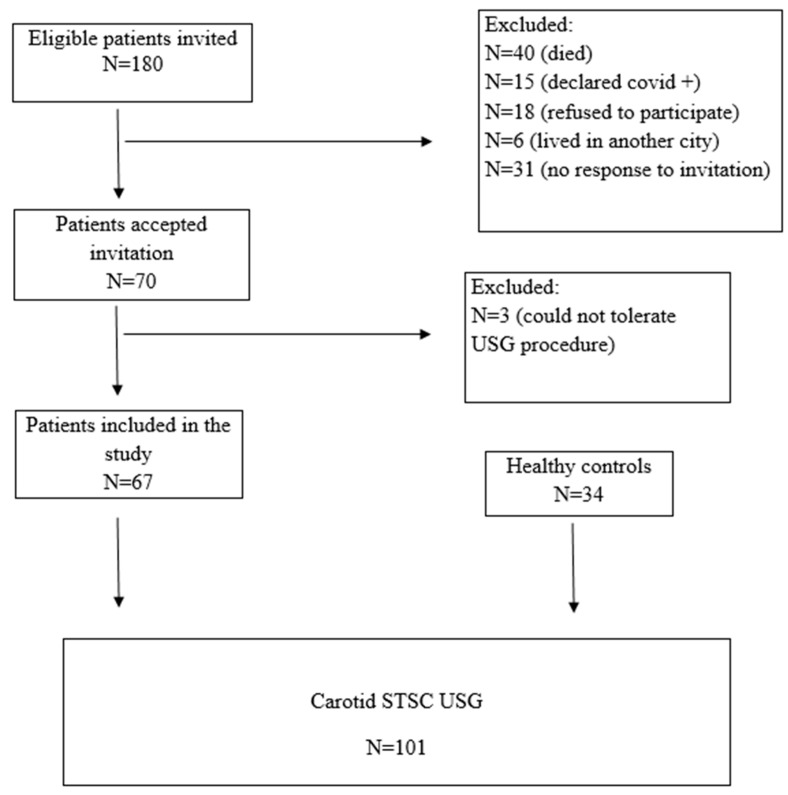
Consort flow diagram for patient inclusion and exclusion.

The control group of this study consisted of 34 healthy volunteers without any neck pathology or history of radiotherapy who were presented to the Department of Radiology of Aydın Adnan Menderes University Hospital for other reasons and with similar demographic characteristics. Written informed consent was obtained from each individual who participated in this study. A CONSORT flow diagram for patient inclusion is summarized (Figure 1).

Participants were grouped as follows.

Group 1: Neck-irradiated patients without neck dissection (IR+/ND−) (28 patients).
Group 2: Neck-irradiated patients with neck dissection (IR+/ND+) (24 patients).
Group 3: Non-neck-irradiated patients with neck dissection (IR−/ND+) (15 patients).
Group 4: Non-neck-irradiated controls without neck dissection (IR−/ND−) (34 controls).

All participants underwent STCS-US under COVID-19 pandemic precautions. Body mass indexes were calculated. Before the STCS-US, subjects were rested in supine positions, in dim lighting, and at an ideal room temperature for a minimum of 10 min. Then, the systolic blood pressure (SBP) and diastolic blood pressure (DBP) were measured with a pulse wave analysis of the brachial artery using a Riester sphygmomanometer (Riester 1312 Minimus II, Rudolf Riester GmbH, Jungingen, Germany). The SBP and DBP were entered into the software (V3.01.09.0315). Participants were also asked about their medical histories in relation to hypertension, diabetes mellitus, stroke, smoking, and hypercholesterolemia. Participants’ entries were revisited for their demographic data, primary malignancies, presence of neck dissection, and radiation therapy.

The study design and protocol were approved by Aydın Adnan Menderes University, Medical Faculty, Non-interventional Clinical Trials Ethics Committee (Trial Protocol Number: 2021/65). Each stage of this study follows the articles of the Declaration of Helsinki.

### 2.2. Speckle Tracking Carotid Strain Analysis (STCS)

All participants underwent STCS-US between March 2021 and April 2021 to evaluate carotid arterial stiffness and strain parameters. Increases in elastic modulus, β-stiffness index, carotis media thickness (CIMT), and PWV indicate an increase in arterial stiffness. These increases in arterial distensibility, compliance, displacement, strain, and strain rate correspond to more significant arterial distension per unit pressure [16].

Arterial stiffness parameters were measured by an experienced radiologist (with 15 years of experience in ultrasound) in the Radiology Department. Carotid B-mode ultrasound was applied with a high-resolution B-mode device (Samsung Medison RS80, Seoul, Republic of Korea) using an L3-12A (Samsung Medison Co., Ltd., Seoul, Republic of Korea) linear probe. The evaluations included both carotid arteries in the head midline and hyperextension positions. Both common carotid arteries (CCA) were evaluated using recordings at ≥2 consecutive beats.

All carotid strain and stiffness parameters were measured using Arterial Analysis Software (Samsung Medison Co., Ltd.). Vascular displacement was calculated automatically, and the software (V3.01.09.0315) evaluated the functional capabilities of the carotid artery. The analysis location was 5–10 mm below the carotid bifurcation. The user defined the control points in the vascular structure wall. Then, the software (V3.01.09.0315) automatically followed the optical flow algorithm in a determined frame. The control points were limited to maintaining the round shape of the vessel and moving within a specific focal range. The tracking suitability was confirmed by the software (V3.01.09.0315) through two trials.

**Figure 2:**
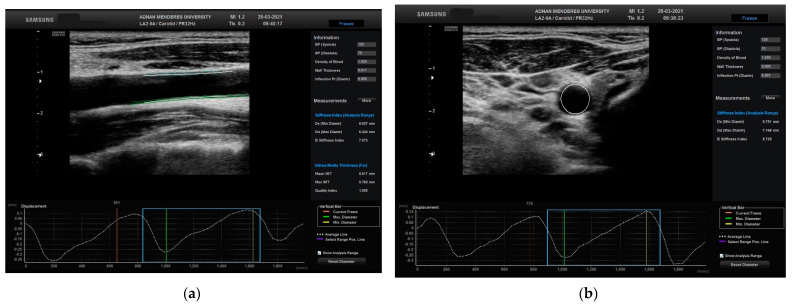
Evaluation of the right CCA in a normal patient in (**a**) longitudinal plane with related automated CIMT and displacement measurements and (**b**) transverse plane with related automated CIMT and displacement measurements. CCA: common carotid artery; CIMT: carotid media thickness.

The movement of the CCA was calculated in two different planes, longitudinal and transverse, using STCS analysis (Figure 2).

The mean value of the CIMT was semi-automatically calculated for each CCA in the longitudinal plane at a location where the non-tortuous region is a minimum of 5–10 mm below the CCA bulb roots. Both the anterior and posterior wall interfaces that define the blood-intima boundaries in the carotid artery (at least four spots in all) were marked on a still image. The movement of the scored points was automatically monitored using the software (V3.01.09.0315). The averages of the right and left carotid artery CIMT values were used in the study analysis.

Arterial stiffness parameters, such as elastic modulus (EM), arterial distensibility (AD), arterial compliance (AC), β-stiffness index (β-SI), and pulse wave velocity (PWV), were calculated using radial measurements and arterial analysis software (V3.01.09.0315). To calculate the arterial stiffness parameters, a trained, experienced technician measured the systolic and diastolic blood pressure with pulse wave analysis of the brachial artery using the Honsun (Nantong) Co., Ltd., Nantong, China sphygmomanometer. Blood pressure measurements were performed on the antecubital region after the patients had rested in the supine position for a few minutes. The measured systolic and diastolic blood pressures were entered into the software (V3.01.09.0315), and all arterial stiffness parameters were automatically calculated. An increase in EM, β-SI, and PWV indicates an increase in arterial stiffness. In contrast, increases in AD and AC mean more significant arterial distension per unit pressure.

### 2.3. Statistical Analysis

The Kolmogorov–Smirnov test was used to assess the normality of numeric variables. For the numeric variables that were normally distributed, intergroup comparisons were made using a one-way ANOVA test, and descriptive statistics were presented as mean ± standard deviation. The Kruskal–Wallis test compared four groups for the numeric variables that were not normally distributed, and descriptive statistics were presented as the median (25–75 percentiles). The Chi-square test was used to analyze the categorical data, and descriptive statistics were presented as the frequency (%); *p* < 0.05 was considered statistically significant.

## 3. Results

A total of 101 participants were included in the study, consisting of 34 controls and 67 patients. The gender distribution among the patients was that 79.10% were male (53 individuals) and 20.89% were female (14 individuals). In contrast, among the controls, 73.52% were male (25 individuals) and 26.47% were female (9 individuals). The median age for the controls was 64.17 years and for patients it was 66 years.

Regarding the primary tumor location, the patient group showed uneven distribution, with the majority having tumors in the larynx (29, 43.28%), followed by the hypopharynx (4, 5.97%), oropharynx (2, 2.98%), oral cavity (17, 25.37%), lip (7, 10.44%), and other locations (8, 11.94%). In terms of radiotherapy, 52 patients (77.61%) were irradiated at least two years before enrollment into this study. The median radiotherapy dose for patients was 66 Gy.

Regarding the TMN stages of the participants, 19 patients (28.35%) had T1, 21 patients (31.34%) had T2, 20 patients (29.85%) had T3, and 75 patients (10.445) had T4 tumors. Nodal involvement was distributed as 49 patients (73.13%) had N0, 7 patients (10.44%) had N1, and 11 patients (16.41%) had N2. There were no patients with N3 involvement. Also, there were no metastatic patients.

Regarding participant comorbidities, hypertension was observed in 9 controls (26.47%) and 15 patients (22.38%), resulting in 24 participants (23.76%) with this comorbidity. Diabetes mellitus type II was present in 9 controls (26.47%) and 10 patients (14.92%), totaling 19 participants (18.81%). Hyperlipidemia was reported in 1 control (2.94%) and 1 patient (1.49%), totaling 2 participants (1.98%). Smoking was reported by 17 controls (50.00%) and 35 patients (52.23%), totaling 52 participants (51.48%). Lastly, no controls experienced a stroke, but 3 patients (4.47%) had a history of stroke, resulting in up to 3 participants overall with this comorbidity (2.97%) (Table 1).

Each neck was evaluated separately because the treatment might have differed for the right and left necks. Group (IR+/ND−) consisted of 28 patients and 56 necks, all treated bilaterally. A total of 32 necks, 8 bilateral, 7 right, and 9 left were included in 24 patients in Group (IR+/ND+). There were 19 necks in 15 patients in Group (IR−/ND+), including 4 bilateral, 4 left, and 7 right neck dissections. The necks of 34 healthy controls were evaluated on both sides; thus, 68 necks were included. Therefore, a total of 175 necks were included in the study. The patients were treated with a median radiation dose of 66 Gy (range: 44–72) in the neck.

The comparison of stiffness and strain parameters between groups revealed exciting associations. Transverse arterial stiffness parameters (AC, EM, and PWV) were statistically significantly changed in the control group compared to Group (IR+/ND−) and Group (IR+/ND+) (*p* < 0.001 for all three parameters, respectively). β-SI was significantly increased in Group (IR+/ND+), and intergroup comparisons with Group (IR−/ND+) and the control group were statistically significant (*p* = 0.002) (Table 2).

Intergroup comparisons of radial strain parameters revealed a statistically significant difference for Group (IR+/ND+) compared to the control group and Group (IR−/ND+) for displacement (*p* = 0.001). Strain and strain rates were significantly reduced in Group (IR+/ND+) compared to Group (IR−/ND+) and Group (IR+/ND−) (*p* = 0.001 and *p* = 0.005, respectively). As for circumferential strain parameters, displacement in Group (IR+/ND+) was significantly different compared to the control group and Group (IR-/ND+) (*p* = 0.001). Circumferential strain and strain rate differences in Group (IR+/ND+) were also significant compared to Group (IR−/ND+) and Group (IR+/ND−) (*p* = 0.001 and *p* = 0.003, respectively). This finding indicates that neck irradiation alone affects circumferential strain parameters to a greater extent than neck dissection alone. Statistically significant changes in Group (IR+/ND+) emphasize the impact of the combined effects of dissection and irradiation (Table 2).

Comparison of the CIMT, CIMT quality index (QI), stiffness, and strain parameters in the longitudinal plane revealed that the effects of irradiation and dissection are similar in all groups compared to the control group. The quality index CIMT produced did not make much difference among the groups, and because this value was close to 1, the accuracy of CIMT measurements was confirmed. Moreover, the difference was statistically significant when irradiated and neck dissected patients were compared to the controls (*p* = 0.001, *p* = 0.021, respectively). As for the stiffness parameters, β-SI was lowest in the control group and highest in Group (IR+/ND+). Therefore, the intergroup difference was statistically significant (*p* = 0.006). Irradiation and dissection increased the stiffness of the arteries. AC was significantly increased in the control group compared to Group (IR+/ND-) and Group (IR+/ND+). (*p* < 0.001).

Moreover, AC was significantly reduced in Group (IR+/ND+). AD was significantly increased in the control group. The difference between the groups for AD was statistically significant for the control group (*p* < 0.001). Both irradiation and neck dissection caused a significant decrease in AD. The comparisons for EM were also significant for the control group. Both irradiation and dissection severely harmed the elasticity of the arteries (*p* < 0.001). This change caused an increase in PWV, and intergroup comparison was significant for the control group (*p* < 0.001). Among radial strain parameters, only displacement was significantly reduced in Group (IR+/ND+) compared to Group (IR−/ND+) and the control group (*p* = 0.012) (Table 3).

## 4. Discussion

Our study investigated the impact of neck dissection and radiotherapy on the arterial stiffness parameters of neck-irradiated patients with neck dissection and without neck dissection and compared the results with healthy controls. Nasopharyngeal carcinoma cases were excluded from the study population, as surgery (i.e., neck dissection) is not a primarily recommended treatment modality for this subset of patients [19]. It aims to fill the research gap in understanding the specific effects of neck dissection on arterial stiffness, as most studies have primarily examined the effects of radiotherapy [6,8,9,10,11].

In the general setting, Group (IR+/ND+) and Group (IR+/ND−) differed from the control group regarding arterial stiffness, radial strain, and circumferential strain parameters in both transverse and longitudinal planes. In contrast, the Group (IR−/ND+) and control group were similar regarding these parameters. For this reason, we considered that neck dissection alone does not increase arterial stiffness. Irradiation following neck dissection statistically significantly increased arterial strain and strain rate parameters compared to irradiation without neck dissection. This result suggests that the manipulations around the tissues of the carotid artery during neck dissection make it more vulnerable to arterial stiffness. The current study contributes to this body of knowledge by demonstrating that the combination of neck dissection and radiotherapy leads to increased arterial stiffness compared to either treatment alone.

As expected, in our study, the transverse radial and transverse circumferential arterial strain and strain rate were lower in Group (IR+/ND+) compared to the control group, Group (IR+/ND−), and Group (IR−/ND+) because of the combined effects of neck dissection and radiotherapy in increasing arterial stiffness. Several studies with STCS have been performed on carotid arterial stiffness in head and neck cancer patients treated with radiotherapy [20,21,22]. In one of these, when the control and lymphoma patient groups’ pulse wave velocity and distensibility values were compared, the arterial stiffness was determined to increase after radiotherapy, especially above the age of 35 [21]. In another study with 50 head and neck cancer patients, when the neck side receiving radiotherapy and the opposite side not receiving radiotherapy were compared for arterial stiffness parameters, no significant difference was found in the β-SI, whereas the EM value was increased. Surgery was found not to make any difference in arterial stiffness [7]. In our study, these two values were statistically significantly higher in Group (IR+/ND−) and Group (IR−/ND+) compared to the control group ((transverse β-SI *p* < 0.001, *p* = 0.017), (longitudinal EM *p* < 0.001, *p* = 0.008)). Moreover, no difference was found in other comparisons except in the EM value with the control group. The transverse β-stiffness index value was significantly higher in Group (IR+/ND+) than in Group (IR−/ND+) alone; however, no significant difference was found compared to Group (IR+/ND−).

In a cohort study investigating the increased risk of stroke after neck dissection, 5827 head and neck cancer patients who underwent neck dissection were recruited. It has been found that neck dissection increases the risk of stroke in patients with more than two risk factors for carotid artery stenosis [23]. Again, in the same study, the risk of stroke was higher in patients with bilateral neck dissection than those with unilateral neck dissection, indicating that the risk of stroke depends not only on the patient’s risk factors but also on surgical manipulation [23]. In our study, the Group (IR−/ND+) was not different from the control group. However, the arterial strain-increasing effect of radiotherapy was higher in Group (IR+/ND+) when compared to Group (IR+/ND−). Considering this information, neck dissection should be performed more carefully in patients with head and neck cancer, especially if they have cardiovascular risk factors.

Furthermore, another study indicating that cerebrovascular risk might be higher due to carotid artery manipulation during neck dissection stated that movements such as carotid artery hyperextension or rotation might induce intimal separation and thrombus. The retraction of the artery might also cause a detachment of the thrombus. This review reported a stroke risk of 4.8% in head and neck cancer patients following neck dissection and 4.8% in head and neck cancer patients [24]. Therefore, considering the risks related to carotid artery stiffness and strain secondary to neck dissection and subsequent radiotherapy, neck Doppler ultrasound and measurements of stiffness parameters are recommended to closely monitor stroke risk in head and neck cancer patients [25].

Overall, our study should be evaluated within its limitations. The variability in the study group, such as different locations of primary tumors and different stages of tumors during initial oncologic treatment, makes it difficult to establish a concrete protocol for neck dissection. More extensive prospective studies are needed to establish surgical protocol and irradiation techniques.

Another limitation of this study is that two-thirds of the patients had N0 neck, and patients with advanced nodal disease were underrepresented. Sixty per cent of the node-dissected patients did not receive radiotherapy for their necks. Patients with an advanced nodal disease are more likely to have chemoradiotherapy and/or extensive nodal dissection, which would affect not only results but also treatment outcomes. A more balanced nodal distribution of patients would have provided more robust conclusions regarding the effects of node dissection and radiation neck treatment in assessing arterial stiffness.

## 5. Conclusions

In conclusion, this study indicates that radiation and surgical treatment of the neck are associated with higher arterial stiffness than single treatment with surgery and radiation. Furthermore, a single treatment is associated with higher arterial stiffness than the control group without treatment. Therefore, radiation and surgery significantly affect arterial stiffness, and double treatment is associated with higher arterial stiffness than a single treatment. Our findings also show that a single treatment with irradiation affects strain parameters more than neck dissection alone. Incorporation of carotid B-mode ultrasound and arterial stiffness parameters into routine clinical practice would benefit patients with head and neck cancer in cardiovascular risk assessment.

## Figures and Tables

**Table 1:** Baseline characteristics of the study cohort.

	Controls (*n* = 34)	Patients (*n* = 67)	Total (*n* = 101)
**Gender**			
Male	25 (73.52%)	53 (79.1%)	78 (77.22%)
Female	9 (26.47%)	14 (20.89%)	23 (22.77%)
**Age (years)**			
Median	64.17	66	64
**Primary Tumor Location**			
Larynx	-	29 (43.28%)	29 (28.43%)
Hypopharynx	-	4 (5.97%)	4 (3.92%)
Lip	-	7 (10.44%)	7 (6.93%)
Oropharynx	-	2 (2.98%)	2 (1.98%)
Oral cavity	-	17 (25.37%)	17 (16.83%)
Others	-	8 (11.94%)	8 (7.92%)
**Radiotherapy Treatment**	-	52 (77.61%)	52 (77.61%)
**Radiotherapy Dose (Gy)**			
Median	-	66	66
**T Stage**			
T1	-	19 (28.35%)	19 (18.62%)
T2	-	21 (31.34%)	21 (20.58%)
T3	-	20 (29.85%)	20 (19.60%)
T4	-	7 (10.44%)	7 (6.86%)
**N Stage**			
N0	-	49 (73.13%)	49 (48.51%)
N1	-	7 (10.44%)	7 (6.93%)
N2	-	11 (16.41%)	11 (10.89%)
N3	-	0 (0%)	0 (0%)
**M Stage**			
M0	-	67	67
**Hypertension**	9 (26.47%)	15 (22.38%)	24 (23.76%)
**Diabetes mellitus**	9 (26.47%)	10 (14.92%)	19 (18.81%)
**Hyperlipidemia**	1 (2.94%)	1 (1.49%)	2 (1.98%)
**History of stroke**	-	3 (4.47%)	3 (2.97%)
**Smoking**	17 (50%)	35 (52.23%)	52 (51.48)

**Table 2:** Comparison of the stiffness and strain parameters in the transverse plane between the groups.

Parameters	IR+/ND− (*n* = 56)	IR+/ND+ (*n* = 32)	IR−/ND+ (*n* = 19)	IR−/ND− (*n* = 68)	*p*-Value
**Stiffness parameters**					
β-SI	8.41 (5.84–12.72)	10.67 (7.41–14.01) ^€^	8.35 (5.33–10.06)	6.75 (6.00–8.60) ^¥^	**0.002**
AC (mm/kPa)	0.59 (0.39–0.86)	0.50 (0.28–0.75) ^€^	0.76 (0.54–1.18)	0.76 (0.59–1.14) ^¥^	**<0.001**
EM (kPa)	107.36 (82.43–174.06)	154.25 (98.33–215.11)	119.29 (74.02–176.85)	89.17 (74.74–108.72) ^¥^	**<0.001**
PWV (m/s)	6.37 (5.76–7.98)	7.44 (6.23–8.96)	6.51 (5.20–8.26)	5.83(5.33–6.36) ^¥^	**<0.001**
**Strain parameters (radial)**					
Displacement (mm)	0.36 (0.29–0.51)	0.34 (0.24–0.44) ^€^	0.41 (0.33–0.62)	0.43 (0.37–0.51)	**0.001**
Strain (%)	5.54 (4.50–7.93)	4.95 (3.45–5.86) **	6.51 (4.74–8.79)	6.28 (5.59–7.45) ^¥^	**0.001**
Strain rate (1/s)	0.63 (0.49–0.89)	0.53 (0.40–0.69) **	0.79 (0.57–0.95)	0.68 (0.61–0.81) *	**0.005**
**Strain parameters (circumferential)**					
Displacement (mm)	0.048 (0.038–0.067)	0.045 (0.030–0.056) ^€^	0.055 (0.043–0.081)	0.055 (0.048–0.066)	**0.001**
Strain (%)	5.61 (4.36–7.90)	4.75 (3.39–5.75) **	6.37 (4.68–8.87)	6.17 (5.52–7.44) *	**0.001**
Strain rate (1/s)	0.65 (0.51–0.87)	0.55 (0.40–0.68) **	0.76 (0.60–0.92)	0.67 (0.60–0.80) *	**0.003**

Values are presented as means ± standard deviation. * There is a statistical difference between the (IR−/ND−) and (IR+ND+) groups. ** (IR+/ND+) group is statistically different from the (IR−/ND+) and (IR+/ND−) groups. ^¥^ (IR−/ND−) group is statistically different from the (IR+/ND−) and (IR+/ND+) groups. ^€^ (IR+/ND+) group is statistically different from the (IR−/ND−) and (IR−/ND+) groups.; β-SI: β-stiffness index; AC: arterial compliance; EM: elastic modulus; PWV: pulse wave velocity.

**Table 3:** Comparison of the carotid intimal thickness (CIMT), CIMT quality index (QI), stiffness, and strain parameters in the longitudinal plane between the groups.

Parameters	IR+/ND− (*n* = 56)	IR+/ND+ (*n* = 32)	IR−/ND+ (*n* = 19)	IR−/ND− (*n* = 68)	*p*-Value
CIMT mean	0.92(0.76–1.11)	0.83(0.69–1.10)	0.82(0.72–0.86)	0.74 (0.64–0.88) *	**0.001**
CIMT QI	0.82(0.68–1.00)	0.90(0.64–1.00)	1.00(0.85–1.00)	1.00(0.79–1.00) *	**0.021**
**Stiffness parameters**					
β-SI	7.74 (5.52–10.53)	8.74 (6.09–10.14)	7.26 (5.35–10.01)	6.92 (4.95–8.26) *	**0.006**
AC (mm/kPa)	0.55 (0.41–0.76)	0.47 (0.33–0.65) ^€^	0.67 (0.42–1.30)	0.71 (0.58–1.06) ^¥^	**<0.001**
AD (/kPa)	0.010 (0.007–0.015)	0.008 (0.007–0.011)	0.010 (0.007–0.014)	0.012 (0.009–0.016) ^¥^	**<0.001**
EM (kPa)	104.68 (66.51–143.56)	124.05 (89.66–150.80)	95.37 (65.47–137.41)	86.6 (62.48–106.09) ^¥^	**<0.001**
PWV (m/s)	6.17 (4.97–7.32)	6.85 (5.59–7.46)	6.07 (5.22–7.13)	5.7 (4.86–6.27) *	**<0.001**
**Strain parameters (radial)**					
Displacement (mm)	0.37 (0.32–0.52)	0.37 (0.25–0.46) ^€^	0.49 (0.31–0.79)	0.44 (0.36–0.55)	**0.012**
Strain (%)	6.68 (5.06–8.69)	5.65 (4.33–7.58)	7.33 (5.22–9.07)	6.86 (5.81–8.51)	0.093
Strain rate (1/s)	0.61 (0.41–0.84)	0.63 (0.50–0.78)	0.71 (0.47–0.86)	0.72 (0.62–0.84)	0.093

Values are presented as means ± standard deviation. * There is a statistical difference between the (IR−/ND−) and (IR+ND+) groups. ^¥^ (IR−/ND−) group is statistically different from the (IR+/ND−) and (IR+/ND+) groups. ^€^ (IR+/ND+) group is statistically different from the (IR−/ND−) and (IR−/ND+) groups. CIMT: circumferential intima-media thickness; β-SI: β-stiffness index; AC: arterial compliance; AD: arterial distensibility; EM: elastic modulus; PWV: pulse wave velocity.

## Data Availability

Regarding availability of the data and materials, if you need clarifications or additional information about the study data, you can email mustafagok@yahoo.com.
